# Mr. Potts, on Artificial Legs

**Published:** 1801-11

**Authors:** I. Potts


### *To the Editors of the Medical and Phyfical Journal.*

Gentlemen,

It is but a few days fince I faw in your Journal for May, 1801, a letter from Mr. T. R. Reid; to which, as it contains a very peculiar attack on myfelf, I hope you will permit me to reply.

When it is known that Mr. Reid *was*, *if he is not still*, the accredited agent for the fale of Mr. Mann's artificial leg in London and its vicinity, the *insidious affectation of candour that glares through his letter may not meet with all the credit he would wish*; however this may be, I fhall fairly and openly difcufs the queftion he feems defirous to agitate.

I was miftaken when I afferted, that "till the appearance of my invention, no artificial leg had been produced, in which the perfon ufing it was not obliged to make a femi-circular motion in walking." I take no fhame to myfelf in making this confeffion, becaufe, when I did make the affertion, I fpoke truth, according to the beft of my knowledge. I had feen no fuch invention, I had not heard of any; my own misfortune alone

(1.) Second Voyage of Captain Cook, Vol. IV.

(2.) Virey Effai d'Hiftoire Naturelle et Phyfiolog. fur la Perfectibilité de l'Homme. Paris, in 8vo.

ſtimulated me to exert my own ingenuity, and produce that which I have now offered to the public; it has been ſeen and approved by many gentlemen of profeſſional eminence, and this it is which has really excited the fears of Mr. Reid; not for himſelf, indeed, but with the feelings of a faithful ſteward, for the intereſts of his employer.

Under the pretence of correcting *my mistake* he introduces a long extract from Mr. Mann's Deſcription of his Patent Leg, which is producing Mr. M's ipſe dixit for the merit of his own invention; this is followed by a commendatory certificate from ſeveral profeſſional gentlemen, and concludes with Mr. Reid's, the *agent*, aſſertion, that Mr. Mann, his principal, FIRST diſcovered and introduced to public notice, an inſtrument ſo capable of relieving one of the greateſt calamities incident to humanity.

If this is not the puff direct for his employer, *artfully introduced under the pretence of correcting my mistake*, I have greatly miſunderſtood it; upon this ſubject, however, I ſhall only remark that Mr. Reid has himſelf committed the very *mistake* which he writes to correct in me, viz. he aſſerts that Mr. Mann FIRST diſcovered an inſtrument ſo capable of alleviating one of the greateſt calamities incident to humanity.

That the date of Mr. Mann's invention was prior to mine I know, I know too that others were made before the date of his, that poſſeſſed the ſame property of bending without obliging the wearer to make the ſemi-circular motion in walking. I do not ſay they were *like* mine or Mr. Mann's, nor ſhall I enquire whether they were equal or inferior to either of ours, but I ſimply acknowledge my conviction that ſuch things were, conſequently both Mr. Reid and I were miſtaken; I, when I aſſerted that *I was the first*; he, when he ſaid *Mr. Mann was the first who invented an instrument possessing such properties.*

I am, &c. I. POTTS.

P. S. Will you allow me to add a letter that I have received from a gentleman whoſe ſituation gives him ample means of knowing the fact he relates, and who can have no intereſt in writing as he does, unleſs it is to ſerve the cauſe of truth; it will place this matter in its proper point of view.

